# Heart Rate Variability in the Perinatal Period: A Critical and Conceptual Review

**DOI:** 10.3389/fnins.2020.561186

**Published:** 2020-09-25

**Authors:** Marco Chiera, Francesco Cerritelli, Alessandro Casini, Nicola Barsotti, Dario Boschiero, Francesco Cavigioli, Carla G. Corti, Andrea Manzotti

**Affiliations:** ^1^Research and Assistance for Infants to Support Experience Lab, Foundation Center for Osteopathic Medicine Collaboration, Pescara, Italy; ^2^Research Commission on Manual Therapies and Mind-Body Disciplines, Societ Italiana di Psico Neuro Endocrino Immunologia, Rome, Italy; ^3^BioTekna – Biomedical Technologies, Venice, Italy; ^4^Neonatal Intensive Care Unit, “V. Buzzi” Children’s Hospital, Azienda Socio Sanitaria Territoriale Fatebenefratelli-Sacco, Milan, Italy; ^5^Pediatric Cardiology Unit-Pediatric Department, Azienda Socio Sanitaria Territoriale Fatebenefratelli-Sacco, Milan, Italy; ^6^Research Department, SOMA, Istituto Osteopatia Milano, Milan, Italy

**Keywords:** autonomic nervous system, vagus, newborns, preterm infants, neonatology, NICU, photoplethysmography, HRV

## Abstract

Neonatal intensive care units (NICUs) greatly expand the use of technology. There is a need to accurately diagnose discomfort, pain, and complications, such as sepsis, mainly before they occur. While specific treatments are possible, they are often time-consuming, invasive, or painful, with detrimental effects for the development of the infant. In the last 40 years, heart rate variability (HRV) has emerged as a non-invasive measurement to monitor newborns and infants, but it still is underused. Hence, the present paper aims to review the utility of HRV in neonatology and the instruments available to assess it, showing how HRV could be an innovative tool in the years to come. When continuously monitored, HRV could help assess the baby’s overall wellbeing and neurological development to detect stress-/pain-related behaviors or pathological conditions, such as respiratory distress syndrome and hyperbilirubinemia, to address when to perform procedures to reduce the baby’s stress/pain and interventions, such as therapeutic hypothermia, and to avoid severe complications, such as sepsis and necrotizing enterocolitis, thus reducing mortality. Based on literature and previous experiences, the first step to efficiently introduce HRV in the NICUs could consist in a monitoring system that uses photoplethysmography, which is low-cost and non-invasive, and displays one or a few metrics with good clinical utility. However, to fully harness HRV clinical potential and to greatly improve neonatal care, the monitoring systems will have to rely on modern bioinformatics (machine learning and artificial intelligence algorithms), which could easily integrate infant’s HRV metrics, vital signs, and especially past history, thus elaborating models capable to efficiently monitor and predict the infant’s clinical conditions. For this reason, hospitals and institutions will have to establish tight collaborations between the obstetric, neonatal, and pediatric departments: this way, healthcare would truly improve in every stage of the perinatal period (from conception to the first years of life), since information about patients’ health would flow freely among different professionals, and high-quality research could be performed integrating the data recorded in those departments.

## Introduction

The neonatology field is growing in complexity (Biban, 2010). In essence, newborns can show many comorbidities associated with prematurity (WHO, 2016), labor complications (Tribe et al., 2018), and maternal and perinatal stress (Frasch et al., 2007; Babenko et al., 2015; Lobmaier et al., 2020), whereas the neonatal intensive care units (NICUs) are increasing the use of technology to better take care of fetuses, newborns, and infants (Biban, 2010; Chock et al., 2015).

However, many obstacles need to be overcome to efficiently assess and manage infants’ conditions: distress, pain, and sepsis need valid and reliable gauges to detect them before they happen (Cremillieux et al., 2018; Rashwan et al., 2019), but several procedures may be time-consuming (Jeng et al., 2000; Als et al., 2005; Cremillieux et al., 2018), invasive, and painful with short- and long-term negative consequences (Holsti et al., 2006; Pillai Riddell et al., 2015).

In the last 40 years, heart rate variability (HRV) has emerged as a reliable and non-invasive measure to monitor preterm and term newborns (Task Force of the European Society of Cardiology the North American Society of Pacing Electrophysiology, 1996). HRV evaluates the heart rate (HR) fluctuation—the variability of the time intervals between successive heartbeats—, and during the years, more and more techniques have appeared to improve its analysis, from which several metrics can be extracted (Table 1; Bravi et al., 2011; Thiriez et al., 2015; Javorka et al., 2017; Oliveira et al., 2019b; Patural et al., 2019).

**TABLE 1:** The most common HRV metrics (Hoyer et al., 2013, 2019; Uhrikova et al., 2015; Pichot et al., 2016; Massaro et al., 2017; Shaffer and Ginsberg, 2017; Herry et al., 2019; Oliveira et al., 2019b; Patural et al., 2019; Frasch et al., 2020).

Metric	Unit	Definition
HR	bpm	HR (number of heart beats per minute)
**Time-domain**		
SDNN	ms	Standard deviation of NN intervals
SDRR	ms	Standard deviation of RR intervals
SDANN	ms	Standard deviation of the average NN intervals for each 5 min segment of a 24 h HRV recording
SDNN Index	ms	Mean of the standard deviations of all the NN intervals for each 5 min segment of a 24 h HRV recording
NNx		Number of adjacent NN intervals that differ from each other by more than x ms (e.g., 5, 20, 25, and 50 ms)
pNNx	%	Percentage of successive NN intervals that differ by more than x ms (e.g., 5, 20, 25, and 50 ms)
RMSSD	ms	Root mean square of consecutive RR interval differences
SDSD	ms	Standard deviation of consecutive RR differences
**Frequency-domain**		
ULF Power	ms^2^	Absolute power of the ultra-low-frequency band (≤0.003 Hz)
VLF Power	ms^2^	Absolute power of the very-low-frequency band (0.0033–0.04 Hz)
LF Peak	Hz	Peak frequency of the low-frequency band (0.04–0.2 Hz for newborns and 0.04–0.15 Hz for infants)
LF Power	ms^2^	Absolute power of the low-frequency band (0.04–0.2 Hz for newborns and 0.04–0.15 Hz for infants)
	Nu	Relative power of the low-frequency band (0.04–0.2 Hz for newborns and 0.04–0.15 Hz for infants) in normal units
	%	Relative power of the low-frequency band (0.04–0.2 Hz for newborns and 0.04–0.15 Hz for infants)
HF Peak	Hz	Peak frequency of the high-frequency band (0.20–2.00 Hz for newborns and 0.20–1.40 Hz for infants)
HF Power	ms^2^	Absolute power of the high-frequency band (0.20–2.00 Hz for newborns and 0.20–1.40 Hz for infants)
	Nu	Relative power of the high-frequency band (0.20–2.00 Hz for newborns and 0.20–1.40 Hz for infants) in normal units
	%	Relative power of the high-frequency band (0.20–2.00 Hz for newborns and 0.20–1.40 Hz for infants)
LF/HF	%	Ratio of LF-to-HF power
VLF/LF	%	Ratio between very-low (0.02–0.08 Hz) and low (0.08–0.2 Hz) frequency band power
LFn		Normalized power in the low-frequency band of the ECG spectrogram (0.04–0.2 Hz for newborns and 0.04–0.15 Hz for infants), i.e., low-frequency power in relation to total power
HFn		Normalized power in the high-frequency band of the ECG spectrogram (0.20–2.00 Hz for newborns and 0.04–0.15 Hz for infants), i.e., high-frequency power in relation to total power
Total power (TP)	ms^2^	Total power of the ECG spectrogram
**Non-linear**		
S	ms	Area of the ellipse that represents total HRV
SD1	ms	Poincaré plot standard deviation perpendicular the line of identity
SD2	ms	Poincaré plot standard deviation along the line of identity
CSI	%	Cardiac Sympathetic Index—SD1/SD2
CVI		Cardiac Vagal Index—log (SD1*SD2)
SVT		Short-term variability of consecutive beat-to-beat data obtained through Poincaré analysis
LTV		Long-term variation of consecutive beat-to-beat data obtained through Poincaré analysis
HRV Triangular Index		Integral of the density of the RR interval histogram divided by its height
HRV Index		Number of all RR intervals divided by the number of RR intervals at the highest point of the RR histogram
TINN	ms	Triangular Interpolation of the NN Interval Histogram—the length of the basis of the minimum square difference of the triangular interpolation for the highest value of the RR histogram or the normalized width of the base of the RR histogram
Parseval Index		Ratio between the square root of the sum of LF and HF powers and the value of SDNN
%DET		Percentage of determinism of a time series from recurrence quantification analysis (RQA): it detects the predictability of dynamical systems
ShanEn		Shannon Entropy—uncertainty of a random variable
ApEn		Approximate entropy, which measures the regularity and complexity of a time series
SampEn		Sample entropy, which measures the regularity and complexity of a time series
MSEx		Multiscale entropy at coarse graining level x
gMSE(x)	bit_norm_	Generalized multiscale entropy at coarse graining level x of NN interval series
QSE		Quadratic Sample Entropy
KLPE		Kullback–Leibler permutation entropy
AC		Acceleration capacity (detection of sequences of two successive RR beats that decrease) obtained through PRSA
DC		Deceleration capacity (detection of sequences of two successive RR beats that increase) obtained through PRSA
ACstx		Acceleration capacity, slope, and step value at coarse graining level x
DFA α1		Detrended fluctuation analysis, which describes short-term fluctuations
DFA α2		Detrended fluctuation analysis, which describes long-term fluctuations
DFA α_S_		Detrended fluctuation analysis, which describes short-term fluctuations
DFA α_L_		Detrended fluctuation analysis, which describes long-term fluctuations
RMS_S_		Root mean square from detrended fluctuation analysis, which describes short-term fluctuations
RMS_L_		Root mean square from detrended fluctuation analysis, which describes long-term fluctuations
SDLE α		Scale-dependent Lyapunov exponent slope
Skewness	a.u.	Skewness of NN interval series
AsymI		Multiscale time irreversibility asymmetry index: it is the degree of temporal asymmetry and lack of invariance of the statistical properties of a signal
D_2_		Correlation dimension, which estimates the minimum number of variables required to construct a model of system dynamics

Several studies showed that HRV correlates with the newborn’s stress and stress-related behaviors (Gardner et al., 2018; Hashiguchi et al., 2020), and that it could predict the baby’s overall wellbeing and future neurological development. HRV could also accurately identify short- and long-term complications, such as the risk of sepsis (Javorka et al., 2017; Oliveira et al., 2019b; Kumar et al., 2020). HRV was also able to reveal the impact of prenatal stress on fetal brain development (Frasch et al., 2007; Lobmaier et al., 2020).

Despite these results, HRV is still underused in NICUs. Although in the 1960s, one of the first evidences published was about the alteration of HRV metrics preceding fetal distress (Task Force of the European Society of Cardiology the North American Society of Pacing Electrophysiology, 1996); to date, some authors argued that there is a lack of understanding of the meaning of HRV metrics in infants: in fact, HRV has been studied especially in adults, and the autonomic nervous system (ANS) behaves differently in newborns, especially in preterm infants (Joshi et al., 2019b). Notwithstanding these contradictions, the only successful example of integration of HRV in NICUs is the Heart Rate Observation (HeRO) monitor developed by J. Randall Moorman’s team. The HeRO analyzes bedside electrocardiogram (ECG) in real-time and integrates various HRV metrics to calculate the “HRC index,” which can predict the risk of sepsis within 24 h in both preterms and very low birth weight infants (Andersen et al., 2019; Kumar et al., 2020).

However, based on the available literature and on the potential research to be developed, there is a need to further explore the use of HRV in neonatology.

HRV may provide such useful insights since it correlates with the ANS development and functioning. The ANS regulates organic development and connects with the organism’s ability to cope with stressors, as well as with cognitive and emotional development (Thayer et al., 2012; Jennings et al., 2015; Schneider et al., 2018; Oliveira et al., 2019b).

To be an innovative tool useful for neonatologists, HRV measurement should rely on a technology that gives reliable metrics with a clear clinical meaning. Harnessing the positive experiences, such as the use of the HeRO monitor, it is paramount to create a system that continuously records HRV and expresses scores that could correlate with the baby’s clinical condition and help monitor its evolution (Zhao et al., 2016; Hayano and Yuda, 2019; Pernice et al., 2019b; Kumar et al., 2020).

For this purpose, modern machine learning (ML) and artificial intelligence (AI) algorithms could play a crucial role: through their computational power, they could define models capable of managing the complex physiological interactions between HRV, ANS, and the whole organism, thus boosting our ability to predict the infant’s prognosis. Indeed, we already have experiences about the clinical usefulness of ML in both neonatology (Semenova et al., 2018; Ostojic et al., 2020) and HRV analysis (Chiew et al., 2019; Lin et al., 2020).

ML/AI algorithms could also integrate clinical data of different hospital departments, i.e., obstetric, neonatal, and pediatric. Indeed, free clinical data and medical devices sharing among the departments involved in the perinatal care (from conception to the first years of life) would allow clinicians to better understand the prenatal and developmental factors underlying adverse neonatal outcomes (e.g., brain injury) and to better treat them.

Therefore, the present paper aims to address the HRV usefulness in neonatology to prospect it as an innovative tool in the years to come. This focused review is divided into three sections: (1) the first section describes briefly the HRV metrics and examines the relationship between ANS and HRV in fetuses and newborns; (2) the second section examines the technology available in the NICU, how to monitor HRV efficiently, and the usefulness of real-time HRV; and (3) the third and final section will summarize the main findings and outline future perspectives for the clinical use of real-time HRV in the neonatal field, with a brief subsection about its usefulness in low-income countries.

## The Ans Physiology Underlying HRV and Its Use in the Neonatal Field

### A Brief Introduction on HRV Metrics

From the work of the Task Force for HRV analysis in 1996, which constitutes the foundation for the majority of the papers on HRV, many metrics are developed to describe HRV, and they can be classified into three categories: time-domain, frequency-domain, and non-linear metrics (Table 1). These metrics can be obtained from short-term (less than 10 min) or long-term ECG recording (24 h) (Task Force of the European Society of Cardiology the North American Society of Pacing Electrophysiology, 1996).

The time-domain metrics are considered to be the simplest methods to extract, since they are directly based on the normal-to-normal (NN) intervals or on the instantaneous HR extracted from the ECG. The time-domain metrics are then calculated through statistical or geometric methods. The statistical methods consist in applying operations, such as mean, standard deviation, or square root, on: the direct NN interval measurements, thus obtaining metrics, such as standard deviation of RR (SDRR), standard deviation of NN (SDNN), standard deviation of the average NN (SDANN), and standard deviation of NN (SDNN) Index, and the difference between NN intervals, thus obtaining metrics, such as root mean square of consecutive RR interval differences (RMSSD), NN50, pNN50, and pNN20 (Task Force of the European Society of Cardiology the North American Society of Pacing Electrophysiology, 1996; Oliveira et al., 2019b; Patural et al., 2019).

The geometric methods consist in transforming the NN interval measurements into a geometric pattern, such as the sample density distribution of the NN intervals (or their difference), from which metrics, such as the triangular interpolation of the NN (TINN), the HRV Triangular Index, and the HRV Index, can be obtained. A Poincaré plot can also be used to plot each NN interval in relation to the previous NN interval and to calculate the standard deviation of the main cluster of data-points, either crosswise (SD1) or lengthwise (SD2) (Task Force of the European Society of Cardiology the North American Society of Pacing Electrophysiology, 1996; Oliveira et al., 2019b; Patural et al., 2019). From the SD1 and SD2 metrics, other metrics, such as Cardiac Sympathetic Index (CSI) and Cardiac Vagal Index (CVI), could be obtained (Oliveira et al., 2019b).

The frequency-domain metrics derive from the analysis of the ECG power spectrum. The power of several frequency bands, in particular ultra-low-frequency (ULF), very-low-frequency (VLF), low-frequency (LF), and high-frequency (HF), are then extracted, as well as other metrics, such as LF and HF normalized indices (LFn and HFn) and the LF/HF ratio. Another frequency index used is the total power (TP), which represents the global variability (Task Force of the European Society of Cardiology the North American Society of Pacing Electrophysiology, 1996; Patural et al., 2019).

Time- and frequency-domain metrics are, however, limited: they could fail in discriminating among different signals that display similar characteristics, such as the same mean or standard deviation. Moreover, ECG recording can manifest several irregularities in the RR series or complex oscillatory phenomena that linear metrics cannot properly describe. Therefore, non-linear analyses were developed to better grasp the complexity behind the brain and ANS influences on HRV (Task Force of the European Society of Cardiology the North American Society of Pacing Electrophysiology, 1996; Bravi et al., 2011). Actually, the geometric methods can be considered a kind of non-linear analysis (Task Force of the European Society of Cardiology the North American Society of Pacing Electrophysiology, 1996; Javorka et al., 2017; Patural et al., 2019).

Several studies showed the usefulness of non-linear metrics over linear ones in evaluating the autonomic development (Uhrikova et al., 2015; de Souza Filho et al., 2019; Oliveira et al., 2019b), the interactions between ANS, brain, and stress axis (Thayer and Lane, 2000), and the infant’s adaptation capacity (Gonçalves et al., 2017; Shaffer and Ginsberg, 2017; Urfer-Maurer et al., 2018).

Many non-linear metrics can be calculated, and every non-linear analysis can be performed at different levels of complexity. Examples are: detrended fluctuation analysis (DFA), sample entropy (SampEn), approximate entropy (ApEn), multiscale entropy (MSE), symbolics dynamics, coarse graining spectral analysis, fractal analysis, and deceleration and acceleration analyses (Task Force of the European Society of Cardiology the North American Society of Pacing Electrophysiology, 1996; Javorka et al., 2017; Patural et al., 2019).

While linear metrics seem more useful when the HR follows periodic oscillations, non-linear metrics, such as SampEn, ApEn, MSE, DFA, and symbolic dynamics metrics, seem more robust to cardiac recording artifacts (Javorka et al., 2017; Stapelberg et al., 2017). Linear and non-linear metrics can, however, detect complementary physiological behaviors (Task Force of the European Society of Cardiology the North American Society of Pacing Electrophysiology, 1996; Bravi et al., 2011): the HRC index, which contains both, represents the best available evidence of their usefulness (Fairchild and O’Shea, 2010).

Actually, many more different techniques of variability analysis are available. To encompass all of them and to refine the original Task Force classification, further classifications have been proposed. Bravi and colleagues, for example, regrouped the various techniques into five domains based on the information they extrapolate: statistical, geometric, energetic, informational, and invariant (Bravi et al., 2011). The present paper, however, will follow the Task Force classification since it still represents the most used.

Although several HRV metrics have been proved useful, few metrics have a precise physiological and clinical interpretation (Table 2). However, we have to consider two crucial limitations: (1) too often HRV is viewed as influenced by only the ANS, with the tendency of reducing a metric to as simply sympathetic or parasympathetic, and (2) although many studies investigate several metrics, the attention is focused mainly on univariate approaches that try to reduce the variables considered at a minimum. This approach could substantially limit the usefulness of the models applied and, thus, the understanding of HRV physiological nature and clinical usefulness (Bravi et al., 2011; Frasch, 2018).

**TABLE 2:** Interpretations and/or usefulness of some HRV metrics (Task Force of the European Society of Cardiology the North American Society of Pacing Electrophysiology, 1996; Shaffer et al., 2014; Shaffer and Ginsberg, 2017; Oliveira et al., 2019b).

Metric	Interpretation
**Most common metrics**	
RMSSD	Main time-domain measure to assess the HRV modulation due to vagal activity
SDNN	Standard metric of overall HRV, influenced by both SNS and PNS. Gold standard for medical stratification of cardiac risk in adults when recorded over a 24 h period
ULF	No consensus regarding the mechanisms underlying ULF power. Very slow-acting biological processes, such as circadian rhythms, are implicated
VLF	Related to the heart’s intrinsic nervous system, which generates VLF rhythm when afferent sensory cardiac neurons are stimulated. SNS activity due to physical and stress responses influences its oscillations amplitude and frequency
LF	Non-specific index that reflects baroreceptor activity, it contains contributions of both the sympathetic and parasympathetic influences
HF	Expression of parasympathetic activity, it corresponds to the HR variations related to the respiratory cycle known as RSA. It changes according to vagal modulation but does not reflect vagal tone
LF/HF	Used to estimate SNS and PNS balance, although LF does not purely represent SNS, and PNS and SNS interact in a complex non-linear manner
**Complex metrics or groups of metrics**	
fABAS	Scale used for evaluating fetal ANS maturation. It derives from the integration of Hoyer et al. (2013): •amplitude (ACTAMP20), evaluating the fluctuation range of heart beat intervals above an approximated baseline increasing complexity; •skewness, evaluating the complexity of heart rate patterns essentially modulated by complex sympatho-vagal rhythms; •gMSE(3), evaluating the asymmetry, contribution of vagal and sympathetic activity with their different time constants, decline of decelerations, and formation of acceleration patterns; •pNN5, evaluating the formation of vagal rhythms; •VLF/LF, evaluating the baseline fluctuation in relation to sympatho-vagal modulations.
DFA α1 – AsymI – KLPE – SDLE α	Used for assessing vagal modulation in fetuses (Herry et al., 2019)
HRC index	Displayed by the HeRO monitor to estimate the risk of sepsis within 24 h. It derives from a logistic regression calculated on standard deviation of the RR intervals, sample asymmetry, and SampEn to detect irregularities and transient decelerations in HR. HRC index is higher in preterm infants than in full term ones (they show less variability), and it decreases as postmenstrual age increases. HRC index can also rise due to acute inflammation, respiratory deterioration, intraventricular hemorrhage, brain injury, NEC, surgery, ventilation, and drugs, such as anticholinergics, anesthetics, and dexamethasone (in this last case, HRC decreases) (Fairchild and O’Shea, 2010; Fairchild, 2013; Kumar et al., 2020)
RMS_S_ – RMS_L_ – DFA α_S_ – LF – HF	Used to estimate outcomes in case of HIE, especially during hypothermia treatment. Low values of RMS_S_, RMS_L_, DFA α_S_, and LF and a high value of HF may predict an adverse outcome and the need of adjuvant neuroprotective therapies. These metrics could discriminate among different types of brain injury. All these metrics decreased also proportionally to NEC severity (Metzler et al., 2017; Al-Shargabi et al., 2018; Campbell et al., 2018)

To overcome these limitations, multivariate analyses were introduced—e.g., continuous individual multiorgan variability analysis (CIMVA) (Seely et al., 2011; Frasch et al., 2014)—, and many researchers started to rely on ML and AI algorithms (Semenova et al., 2018; Vassar et al., 2020). As a consequence, research begun to integrate various metrics—as shown by the HeRO monitor, whose HRC index derives from a logistic regression applied on the standard deviation of the RR intervals, sample asymmetry, and SampEn and aims to detect pathological heart decelerations to predict the risk of sepsis within 24 h (Fairchild and O’Shea, 2010)—, to combine measurements from different organs for predicting clinical conditions (Kumar et al., 2020), and to define models able to describe HRV as a phenomenon influenced by the whole organism (Frasch, 2020).

### The HRV as a Window on the ANS

The ANS plays a central role in homeostasis maintenance and allostatic adaptation, allowing the organism to change its behavior based on the circumstances and stressors, whether internal or external (Thayer and Lane, 2000).

The two ANS branches, the sympathetic (SNS) and parasympathetic system (PNS), continuously modify their balance to finely regulate the organic systems, including the cardiovascular, respiratory, gastroenteric, metabolic, and immune-inflammatory ones (Rees, 2014; Mulkey and du Plessis, 2019). The two ANS branches connect with several brain circuits known as the central autonomic network (CAN), which influences both the ANS and the higher cortical functions, such as cognition control, emotional regulation, and behavior (Benarroch, 1993; Thayer and Lane, 2000).

To cope with the environmental stimuli, the CAN regulates the complex non-linear interaction between SNS and PNS. In the sinoatrial node, this interaction modulates the HRV (Billman, 2011; Shaffer and Ginsberg, 2017). Tools, such as ECG or photoplethysmography (PPG), which monitor cardiovascular activity, can thus evaluate autonomic brain–heart interactions and give information regarding the ANS through HRV metrics (Table 2).

These metrics are usually viewed as revealing different facets of the SNS and PNS activities, with some metrics more related to one of the two branches and the other reflecting more complex ANS, cardiocirculatory, and respiratory activities (Shaffer and Ginsberg, 2017; Oliveira et al., 2019b). However, HRV may reflect much more than the autonomic regulation of cardiac activity. On the one hand, it has been recently discovered, in ovine models, that the fetal heart has already an intrinsic sinoatrial node activity that can affect HRV and that can be affected by adverse conditions (e.g., chronic hypoxia) in the last trimester of pregnancy (Frasch et al., 2020). On the other hand, HRV seems to be greatly influenced by information coming from the whole organism (e.g., the gut or the immune system) through systemic afferent pathways, such as the vagus nerve (Frasch, 2020).

Moreover, HRV may represent an index of the adaptive regulation processes performed by the CAN (Thayer et al., 2009, 2012). Indeed, HRV correlates with CAN activity measured by functional magnetic resonance imaging (Thayer et al., 2012) and several measures of cognitive, emotional, and behavioral regulation (Holzman and Bridgett, 2017; Forte and Casagrande, 2019). Since various HRV metrics showed to be correlated with stress and inflammation, HRV analysis has the potential to give information about the subject’s health (Kim et al., 2018; Williams et al., 2019).

During fetal and neonatal life, the ANS undergoes a prolonged process of development and maturation, during which it remains vulnerable to developmental disruption from a variety of stressful physiological and environmental stimuli. Such stimuli—which can occur during pregnancy (e.g., congenital disease, fetal growth restriction, maternal stress/nutritional deficiency), labor (e.g., premature, complicated, or prolonged birth), and even in the NICU environment (e.g., invasive procedures, loud noise, and bright light)—can significantly influence the developmental trajectory of both the ANS and the CAN. They can reduce the newborn’s capacity to efficiently adapt to a continually changing and challenging environment (Mulkey and du Plessis, 2019).

It is thus essential to know how ANS develops during pregnancy and after birth, and how HRV changes as a result, in order to make efficient use of HRV as an assessment tool.

### The ANS Development and Its Relationship With HRV

Fetal cardiac activity is usually measured through cardiotocography (CTG), which can monitor fetal HR via ultrasound waves (Ayres-de-Campos and Bernardes, 2010). Although able to detect HR accelerations and decelerations, this tool poorly discriminates QRS complexes and is unsuitable to measure fetal HRV (fHRV), especially short-term HRV (Van Leeuwen et al., 2014). Therefore, in the last 20 years, researchers have relied on fetal magnetocardiography (fMCG) and ECG (fECG) to monitor fHRV and assess its correlation with ANS development (Hoyer et al., 2013, 2015).

fECG and fMCG use electrophysiological mechanisms to monitor cardiac activity and better discriminate QRS complexes (Hoyer et al., 2017). fMCG, which is obtained through the spatiotemporal measurement of the heart-related magnetic field, allows a stable and precise HRV assessment during the second and third pregnancy trimesters. However, fMCG is available in a few centers worldwide due to its high cost (Hoyer et al., 2017). fECG can be obtained through electrodes placed on the mother’s abdomen (Hoyer et al., 2017) or directly on the fetal cranium during labor (Warmerdam et al., 2016). fECG performs less accurate measurement and, between 28 and 32 weeks gestational age (GA), is less reliable since the fetus is almost entirely covered by the vernix caseosa, which isolates the fetus hindering the recording (Hoyer et al., 2017). Besides, fECG shows a considerable signal loss (30 ± 24%) with 3.6 ± 1.7 gaps/min, which limits short-term and entropy-related HRV metrics measurements. As a result, fECG shows higher values for these metrics than fMCG (Van Leeuwen et al., 2014).

Using fMCG, Hoyer and colleagues defined the fetal autonomic brain age score (fABAS) (Table 2), which derives from a multivariate analysis of five different metrics—amplitude, gMSE(3), skewness, pNN5, and VLF/LF—and managed to assess ANS development from the 22nd week GA (Hoyer et al., 2013, 2015). Other authors have then proposed to add other metrics to fABAS to define more complete and precise models (Schmidt et al., 2018).

Applying regression models on metrics grouped in short-term and long-term amplitude fluctuations (AMP), complexity (COMP) and pattern categories, Hoyer et al. found that ACst1, STV, MSE4, and skewness were the metrics that best correlated with fetal maturation (Hoyer et al., 2019). During the third trimester, AMP, COMP, and pattern metrics increase, although with different timing (Hoyer et al., 2019), together with sympathetic and parasympathetic modulations. The baseline HR also becomes more regular and with a more stable rhythm (Schneider et al., 2018). As GA increases, even linear metrics—RMSSD, SDNN, LF, and HF—rise as well. These changes indicate the ANS development and better fetal regulatory capacities (Hoyer et al., 2009; Cardoso et al., 2017; Aye et al., 2018).

To achieve an optimal maturation, the ANS, in particular the PNS, needs 37 weeks GA (Patural et al., 2019): indeed, preterm newborns show a sympathetic predominance (Yiallourou et al., 2012). The PNS begins to develop during the first trimester, when the lateral hypothalamus differentiates and the vagus nerve myelination increases (Koutcherov et al., 2003; Cheng et al., 2004). From the 32nd week GA, the vagus nerve regulatory activity increases with the appearance of the baroreflex mechanisms and the respiratory sinus arrhythmia (RSA)—the capacity of HR regulation based on respiratory rate. Meanwhile, fetal movements and HR increase indicate a rise in the SNS activity (DiPietro et al., 1996). The ANS regulatory function, especially the parasympathetic, develops until birth when the ANS has to react to the new environmental conditions by adapting the cardiovascular and respiratory systems during the fetal–neonatal transition (Mulkey and du Plessis, 2018).

At birth, the ANS shows a slight sympathetic predominance—high LF and low HF—, but in the first 24 h after birth, several metrics (e.g., HR, SDNN, CSI, HFn, LFn, TP, SD2, HRV Index, and Parseval Index) change significantly (Oliveira et al., 2019b). In particular, during the first 12 h, the PNS gradually begins to take over, as shown by the LF/HF ratio reduction (Patural et al., 2019).

During the first days and weeks of life, both preterm and term newborns show an increase in RMSSD, SDNN, and HF, although preterm newborns show lower HRV than term ones at the same postmenstrual age: they display lower values of RMSSD, LF, and HF and a higher LF/HF ratio, which likely reflect an ANS that needs more time to mature (Lucchini et al., 2016; Cardoso et al., 2017; Aye et al., 2018).

The ANS, in particular the PNS, continues to develop during the first 2 years of life considerably: indeed, every time, frequency, geometric, and non-linear metric tend to increase (Patural et al., 2019).

Since many HRV metrics change during the ANS development, several authors investigated if specific correlations could be established between those changes and the fetal health status.

According to Hoyer et al. (2019), some AMP and COMP metrics correlate with maternal lifestyle (smoking and physical activity), intrauterine growth restriction (IUGR), and gestational diabetes. Despite the maternal and fetal circulation being distinct systems, HRV fluctuations could represent a coupling mechanism between the two: indeed, maternal HR, TP, and HF correlate with some fHRV metrics, especially during active sleep and when GA > 32 weeks. In particular, maternal HR correlates positively with fHR and negatively with fHRV (Zöllkau et al., 2019).

A different approach based on coupling maternal and fetal HR through phase rectified signal averaging (PRSA) analysis found a correlation between the two HRs, but only for stressed mothers (Lobmaier et al., 2020). A correlation between some fHRV metrics and maternal TP and RMSSD was also found in normotensive mothers, whereas in preeclamptic pregnancies, there was no correlation despite substantial reduction in both maternal HRV (SDNN, RMSSD, pNN50, TP, VLF, LF, HF) and fHRV metrics (SDNN, RMSSD, pNN50, TP, VLF, LF, HF, STV, LTV, N° of accelerations) (Lakhno, 2017).

During labor, although uterine contractions may alter fHRV recording, fetal scalp electrode (FSE) can measure fHRV with adequate precision and give more accurate information about fetal distress than traditional CTG, thus better discriminating between healthy fetuses and those with acidosis (Gonçalves et al., 2006; van Laar et al., 2010, 2011; Spilka et al., 2012; Abry et al., 2013; Georgieva et al., 2013). pH levels during labor were correlated with both linear (Gonçalves et al., 2006; van Laar et al., 2010) and non-linear fHRV metrics (Li et al., 2005; Ferrario et al., 2006; Abry et al., 2013; Chudacek et al., 2014; Signorini et al., 2014), and the combination of linear and non-linear metrics seemed to detect acidosis more precisely than the two types of metrics alone (Spilka et al., 2012; Warmerdam et al., 2018). In addition to FSE, trans-abdominal fECG with a sampling frequency of 900 Hz—way higher than the frequency of 4 Hz used for CTG—may efficiently measure fHRV during labor and predict neonatal pH level and acid–base balance, especially through CIMVA (Frasch et al., 2014; Li et al., 2015). These results are of great significance since acid–base imbalance can be correlated to brain neuroinflammation and, therefore, to the risk of developing brain injury (Xu et al., 2014).

Together with continuous fHRV assessment, the selective measurement and comparison among the fHRV metrics obtained during labor contractions and the ones obtained between successive contractions managed to better detect fetal distress (Warmerdam et al., 2018). Several fHRV metrics increased during contractions and decreased during rest periods (Romano et al., 2006; Cesarelli et al., 2010; Warmerdam et al., 2016), and this variability was lower in fetuses with acidosis than in healthy ones, pointing at a lower cardiovascular adaptation capacity (Warmerdam et al., 2016). Entropy metrics, such as SampEn and ApEn, could also detect fetal distress from the 30th week GA (Ferrario et al., 2006). These two metrics, together with time-domain, complexity (e.g., Lempel Ziv complexity), and PRSA-derived ones, could also distinguish between fetuses with and without IUGR (Signorini et al., 2014).

Non-linear metrics could explain why male fetuses show higher HRV than female but are at higher risk of comorbidity (DiPietro and Voegtline, 2017): while linear metrics are higher in male newborns, non-linear metrics that evaluate entropy are higher in female newborns (Gonçalves et al., 2017; Spyridou et al., 2018). Moreover, non-linear metrics, such as ApEn or MSE, could describe better than linear metrics the ANS development that happens around the last weeks of gestation, when the fetus prepares for birth (Spyridou et al., 2018).

Interesting results came also from animal models of fetal inflammation. Studies on models of hypoxic–ischemic events in ovine fetuses showed that many fHRV metrics changed in the hours after the event: VLF decreased, whereas SampEn, HF, and RMSSD increased (Frasch et al., 2016; Kasai et al., 2019). Besides, the rise in RMSSD correlated positively with interleukin (IL)-1β levels (Frasch et al., 2016), whereas lipopolysaccharides administration showed that multidimensional fHRV metrics (i.e., both linear and non-linear) could predict the fetal inflammatory response (Durosier et al., 2015; Herry et al., 2016).

In an ovine model of repetitive umbilical cord occlusions, PRSA applied to fHRV detected hypoxic events and distinguished between mild, moderate, and severe hypoxia–acidemia (Rivolta et al., 2014). This result was deepened in another study where an ML algorithm based on RMSSD time series sampled at 1,000 Hz was developed: after 2 h of training with the individual fHRV during labor, the algorithm predicted fetal cardiovascular decompensation with 92% sensitivity, 86% accuracy, and 92% precision. When using 4 Hz CTG, the algorithm showed a 67% sensitivity, 14% accuracy, and 18% precision—that is, sample frequency really matters (Gold et al., 2019).

fHRV assessment could thus give precious information both on the physiological fetal maturation and on the occurrence of pathologies that, whenever left unresolved, are able to induce lifelong complications (Hoyer et al., 2017; Frasch, 2018). Assessing HRV in newborns and infants could, thus, greatly improve neonatal care (Kumar et al., 2020). Therefore, a discussion is required about the technology used to measure HRV in newborns and infants and about the physiological or environmental factors that could influence HRV.

## A Technological Analysis to Efficiently Monitor and Use HRV

### The Current NICU Monitoring Instruments

The NICU incubators are closed devices equipped with advanced devices to monitor the newborn’s conditions. In essence, they guarantee a clean and controlled environment concerning temperature, humidity, and even mechanical noise and light. They monitor vital signs through sensors and electrodes placed on the babies (Rajalakshmi et al., 2019).

The foremost vital signs—temperature, blood pressure, pulse frequency, HR, and SpO_2_—are measured through monitors for the control of pressure, cardiorespiratory system, skin temperature, and carbon dioxide and oxygen concentrations; pulse oximetry; and mechanical ventilator. In the case of anomalies, an alarm alerts the NICU staff to intervene promptly (Rajalakshmi et al., 2019).

ECG represents the gold standard to monitor HR calculated through the RR interval (Phillipos et al., 2016; Alonzo et al., 2018). Pulse oximetry can instead measure HR through the pulse rate, but its usefulness is debated: evaluating pulse rate is difficult when peripheral tissue perfusion is reduced (Mizumoto et al., 2012), and the measurement is sensitive to motion artifacts (Sahni et al., 2003).

Pulse oximetry performs a simple, non-invasive, and accurate SpO_2_ measurement to evaluate the risk of hypoxia and/or hyperoxemia, the appropriateness of the oxygen administered to the newborn, and the presence of ductal dependent congenital cardiac diseases (Stenson, 2016; Kumar et al., 2020). Pulse oximetry, however, struggles to detect hyperoxemia when SpO_2_ > 94% (Hay, 1987).

Blood pressure (influenced by cardiac output and peripheral vascular resistance) can be measured through an external cuff, which, however, can underestimate it in hypotensive or pathological preterm newborns (Cunningham et al., 1999). A peripheral arterial catheter can continuously monitor blood pressure, although it can induce severe cardiovascular complications (Werther et al., 2018).

The respiratory rate is calculated through two electrodes placed on the thorax and the abdomen that measure the thoracic impedance changes. Plethysmography gives a more reliable measure but requires a higher number of thoracic electrodes (Weese-Mayer et al., 2000).

Temperature is continuously monitored because preterm newborns struggle to regulate it and can suffer from hypothermia. Incubators can assess the newborn’s temperature through skin sensors and probes or infrared thermography, which has the advantage of being contactless and non-invasive, and may adapt the environmental temperature to the newborn’s skin temperature (Verklan and Walden, 2014). Due to the sensitive skin of newborns, cutaneous sensors could create stress and even damage the skin (Topalidou et al., 2019).

Despite continuous monitoring to assure high care standards, much of this collected information remains unused. This situation is worth noting since integrating these vital signs could improve the predictive power of the NICU monitors and better help reduce infants’ mortality (Sullivan et al., 2018; Joshi et al., 2020; Kumar et al., 2020).

Besides monitoring the infant’s clinical condition, incorporating the infant’s history would also be of the utmost importance. Information about the baby’s fetal health, labor, and past adverse events (e.g., brain injuries) could augment the interpretation of the aforementioned vital signs. In fact, different pathologies in different babies could induce similar alterations in vital signs, and the knowledge of the infant’s history could help recognize what is happening (Kumar et al., 2020).

In this difficult task, NICU monitors based on ML algorithms could be useful for clinicians: integrating the infant’s past and present data, they could discriminate among the several pathologies that could affect the infant, thus pointing at the best preventive and therapeutic strategy. ML algorithms have already helped in predicting the risk of various conditions (e.g., cerebral hemorrhage and hyperbilirubinemia) and in reducing false alarms, thus improving neonatal care (Daunhawer et al., 2019; Malacova et al., 2020; Ostojic et al., 2020; Turova et al., 2020; Vassar et al., 2020).

### The Current State of HRV Assessment and Limitations

The gold standard to evaluate HRV consists of specific calculations on the electrocardiographic HR registrations (time elapsed between two adjacent R peaks) in term and preterm newborns (Kevat et al., 2017).

However, ECG does not seem to be always used properly, and as a consequence, HRV measurement may suffer from methodological issues: for instance, the NICU tools used to monitor HR may have a low sampling frequency (<200 Hz), making the HRV analysis unreliable under several circumstances (e.g., patients with low RR variability due to heart failure), in particular for frequency and non-linear metrics. Some authors indeed advised to use a sampling frequency of at least 500 Hz (Laborde et al., 2017; Shaffer and Ginsberg, 2017). Moreover, in the research field, there is the tendency to use proprietary software without specifying the exact algorithms used to extract the HRV parameters and, thus, limiting the usefulness of the results (Heathers, 2012; Pagani et al., 2012).

Particularly interesting is the case of the frequency-domain metrics, LF and HF. Despite being widely used, the frequency threshold of 0.15 Hz is used to discriminate between them in adults derived from animals, and it seems that it has never been validated in humans (Hayano and Yuda, 2019). Therefore, the frequency-domain metrics could be biased from the beginning: this could be a reason why LF and HF do not reflect a specific physiologic function, but the interaction between PNS, SNS, and respiratory activity (Hayano and Yuda, 2019). Moreover, not all studies on newborns use the same thresholds for discriminating between LF and HF (Andersen et al., 2019).

Among the several HRV metrics (Table 1), few of them have been validated (Table 2; Shaffer and Ginsberg, 2017; Hayano and Yuda, 2019), especially in newborns and infants (Cardoso et al., 2017; Oliveira et al., 2019b). For several metrics, their significance can also change according to the measurement duration: short (about 5 min) or long term (until 24 h) (Pernice et al., 2019b).

Therefore, standardizing how researchers and clinicians should monitor HRV and calculate their metrics is essential. A uniform methodology could also help in defining normative HRV values for infants (stratified for their age and other fetal development characteristics)—during the years, several studies attempted to fill this gap (Clairambault et al., 1992; Eiselt et al., 1993; Spassov et al., 1994; Cardoso et al., 2017; Schneider et al., 2018; Oliveira et al., 2019b; Patural et al., 2019; Shuffrey et al., 2019)—to promptly recognize even deadly complications, but results are still controversial.

As with the integration of the baby’s vital signs and history, clinicians could rely on ML and AI to overcome this difficulty. ML could both define normative HRV values for single or complex metrics and even create models able to predict how the newborn’s condition will evolve based on big-data analysis (Fairchild and Aschner, 2012; Kumar et al., 2020). Indeed, in several research fields including neonatology, neurology, and cardiology, advanced ML is being successfully used to detect specific pathophysiological characteristics (Fraser, 2017; Pinaya et al., 2019; Tu et al., 2020; Turova et al., 2020; Vassar et al., 2020).

### The Use of PPG to Measure HRV as an Alternative to ECG

In the last years, a new methodology that uses PPG has been applied to measure the peripheral pulse rate variability (PRV) through sensors placed on the fingers (Blackford et al., 2016; Choi and Shin, 2017). The growing interest toward PPG rests on its simplicity, low-cost, safety, and minimal invasiveness and on its capacity to assess signs, such as oxygen saturation, and to extract cardiorespiratory parameters (Pernice et al., 2018, 2019a; Singh et al., 2018).

PPG is an optical measurement technology used to detect blood volume changes in peripheral capillaries. It needs a light source and a photodetector that can measure the light intensity variations related to the capillary perfusion changes. PPG is used in physiological and clinical research to measure blood pressure variability (McDuff et al., 2018; Pernice et al., 2018, 2019b).

The absence of electrodes helps reduce the newborn’s stress and pain. It may also decrease the risk of limb ischemia and skin lesions due to electrode-related irritation and, thus, the risk of skin infections with subsequent use of antibiotics and disinfectants, which can alter the cerebral pressure and perfusion when inhaled (Blanik et al., 2016; Zhao et al., 2016; Cobos-Torres et al., 2018). ECG alternatives to monitor HRV are paramount since, due to altered evaporation of body water, electrodes on large skin areas may influence the balance between heat and water in preterm newborns who weigh less than 1,000 g (Blanik et al., 2016). Avoiding electrodes would also reduce the workload of NICU professionals since they might avoid controlling the correct positioning of patches (Cobos-Torres et al., 2018).

From a PPG signal, a PRV time series can be registered and analyzed similarly to HRV (Pernice et al., 2018). However, HRV and PRV are different: HRV is calculated from the cardiac electrical activity, whereas PRV from the mechanics of pulsatile blood. PPG and ECG also show different waveforms: they are differently influenced by the sampling frequency (Choi and Shin, 2017).

PPG recording is affected by physiological factors related to the pulse wave transmission along the vascular bed and by measurement errors, such as artifacts due to movements. In the last years, several authors have attempted to reduce or eliminate these errors that can make PRV and HRV disagree (Pernice et al., 2019b). The PRV analysis should also use a correct sampling frequency to obtain measurements concordant with ECG (Choi and Shin, 2017; Hejjel, 2017).

Some authors found that, for several metrics (SDNN, SDSD, RMSSD, NN50, pNN50, TP, HF, LF/HF, LFn, and HFn), a frequency as low as 25 Hz could give comparable results to those obtained with a 10 kHz-sampled ECG in healthy subjects (Choi and Shin, 2017). However, very low sample frequency could not correctly detect the effect of vagal modulation on HRV/PRV (as it is associated with high frequency), making frequency and non-linear metrics unreliable—time-domain metrics appear instead to be more robust (Choi and Shin, 2017; Béres et al., 2019). Notwithstanding this, other studies used a sample frequency of 200 Hz obtaining reliable results (Sun et al., 2012; Elgendi et al., 2016). Thus it can be argued that the above-mentioned recommendations for ECG could also be valid for PPG.

Most studies consider PRV and HRV to be exchangeable (Blackford et al., 2016; Pernice et al., 2018; Singh et al., 2018), although sometimes different measurements were detected when people (mostly adults) were under postural (45° head-up tilt test) or mental (arithmetic test) stress, especially for RMSSD, LF, HF, and LF/HF (Pernice et al., 2019b).

In the context of NICU, PPG provides accurate data in addition to the ease of use. A recent study showed that, through specific algorithms, PPG could detect the movement artifacts, remove them from the recordings, and use them to evaluate the onset and duration of the newborn’s movements: this measurement could help assess the motor development and even the cognitive growth related to motor control (Zuzarte et al., 2019). These elements could favor the use of PPG over other devices. However, some studies showed lack of sufficient data sampling to perform adequate statistical analysis, calling, therefore, for more robust data collection (Kevat et al., 2017; Henry et al., 2020).

In the last years, non-contact video-photoplethysmography (vPPG) has been introduced: a non-invasive optical technology able to remotely detect the blood volume changes, using natural light. A digital camera measures the little cutaneous light intensity changes, which derive from the cardiovascular rhythm of the skin blood perfusion (Blanik et al., 2016; Valenza et al., 2018). vPPG could accurately monitor the newborn’s movements (Cobos-Torres et al., 2018) and even help prevent sudden infant death syndrome (SIDS) (Zhao et al., 2016).

Since HR tends to significantly decrease over minutes or hours before SIDS occurrence, a technology that could continuously monitor HR, in particular, during night-time or with low ambient light—SIDS happens especially in these conditions—could efficiently prevent SIDS. Using specific algorithms, vPPG seems to make accurate HR measurements during night-time; besides, since vPPG does not rely on skin sensors, it could avoid fatal errors linked to their detachment due to the baby’s movements (Zhao et al., 2016).

From the available evidence on newborns and adults, vPPG and ECG would seem to agree in the detection of HR and several HRV metrics (e.g., LF, HF, SDNN, SampEn, and Lyapunov exponent): the statistical analyses (e.g., Bland–Altman tests and Pearson’s r) showed high correlations, and the measurement errors between the two technologies were similar to those between ECG and other technologies, such as pulse oximeter (Blanik et al., 2014; Villarroel et al., 2014; Blackford et al., 2016; Valenza et al., 2018). Other studies found vPPG and PPG to give similar measurements in the assessment of HR, LF, and HF: since PPG is usually considered as reliable as ECG, the authors inferred that vPPG could also agree with ECG (Sun et al., 2012; Aarts et al., 2013; McDuff et al., 2014; Paul et al., 2020). However, more studies on newborns are needed to improve vPPG and to better evaluate its usefulness (Cobos-Torres et al., 2018; Valenza et al., 2018).

Before proceeding further, we briefly mention ballistography (BSG), another contactless and unobtrusive technology that could help HRV assessment. BSG can detect the mechanical forces exerted by the body, including body movements, breathing motion, and heartbeat, through sensors placed in the bedding or mattress under the infant’s body or even in the incubator rack. From the mechanical signals recorded by the sensors (e.g., including electromechanical film sensors, load-cells, and accelerometers), BSG successfully extrapolated reliable respiratory waveforms and HR recordings in preterm newborns (Nukaya et al., 2014; Lee et al., 2016; Joshi et al., 2018, 2019a). While in neonatology, BSG represents a new technology that should be improved to optimally and precisely detect the weak forces of the baby’s body; in adults, BSG gave reliable HRV measurements as compared with ECG (Shin et al., 2011; Wang et al., 2015).

### Factors That Can Alter HRV Assessment

To optimally use HRV, NICU professionals must be aware there are factors that can influence HRV recording, including the sleep stage, the infant’s position, and the NICU/incubator environment (De Jonckheere and Storme, 2019; Weber and Harrison, 2019).

HRV in newborns is often measured during sleep, which shows two main stages: the quiet sleep (QS), characterized by the absence of movements and slow rhythmic breathing, and the active sleep (AS), characterized by myoclonic twitching, facial, eye, and head movements, and irregular heart and breath rates (Walusinski, 2006; Dereymaeker et al., 2017).

AS and QS show, respectively, a sympathetic and a parasympathetic predominance (Frasch et al., 2007; Yiallourou et al., 2012), with HRV metrics, such as SDNN, LF/HF, LF, and TP higher in AS than in QS. Sometimes, parasympathetic-related metrics were found to be higher during AS than QS (although by a lower degree than the sympathetic-related metrics), maybe to balance the sympathetic activity (Yiallourou et al., 2012; Stéphan-Blanchard et al., 2013; Fyfe et al., 2015; Thiriez et al., 2015; Schneider et al., 2018).

If this were the case, altered parasympathetic-related metrics during AS could reveal that the vagal system and the ANS still have to fully develop (Yiallourou et al., 2012). An immature PNS may, thus, fail to balance the SNS and the hypothalamic–pituitary–adrenal axis activity under stressful conditions, thus increasing the risk of sepsis, necrotizing enterocolitis (NEC), or even SIDS (Yiallourou et al., 2012; Stone et al., 2013; Sullivan et al., 2014; Mulkey et al., 2018).

Indeed, healthy newborns show an increase in several metrics during a head-up tilt test, thus recruiting both ANS branches to counter the hypotensive stressor (Yiallourou et al., 2012). Besides, newborns who later displayed abnormalities, such as altered neurological development (i.e., cerebral palsy, language or mental retardation, vision or hearing disability, or attention disorders) showed altered HRV (lower total and non-harmonic power) just during AS (Thiriez et al., 2015).

Although the studies do not perfectly agree (Fyfe et al., 2015), the prone position correlates with lower SDRR and RMSSD (Galland et al., 2006; Lucchini et al., 2016) and also lower sympathetic tone measured through LF and TP (Jean-Louis et al., 2004): these results could indicate an altered ANS response, possibly clarifying the higher risk of SIDS during prone sleep (Elhaik, 2016). The side position seems to stabilize HR and prevent oxygen desaturation during feeding in newborns with GA < 34 weeks (Thoyre et al., 2012), although a recent study failed to find the same results (Raczyńska and Gulczyńska, 2019).

Concerning the NICU environment, temperature, light, and noise could be a significant source of stress that can affect infants’ HRV (De Jonckheere and Storme, 2019; Weber and Harrison, 2019).

Warm incubators correlated with higher HR, lower parasympathetic activity, HF, and shorter RR intervals (Franco et al., 2000). When the incubator temperature was 2°C lower than the newborn’s skin temperature, the parasympathetic-related metric RMSSD increased in all sleep stages, together with SDNN and HF, whereas the sympathetic-related metric CSI decreased; the opposite findings were obtained when the incubator was 2°C warmer (Stéphan-Blanchard et al., 2013). Therapeutic hypothermia in case of hypoxic–ischemic encephalopathy (HIE) could induce an increase in LF, root mean square from detrended fluctuation analysis, which describes short-term fluctuations (RMS_S_), root mean square from detrended fluctuation analysis, which describes long-term fluctuations (RMS_L_), and DFA α_S_ (Massaro et al., 2017). Extreme variations of environmental or core temperature in both directions can, lastly, destabilize the ANS (as revealed by HRV alteration) and induce possible complications (Fox and Matthews, 1989; Mowery et al., 2011).

Light and sound can alter the newborn’s cardiorespiratory functions, increase stress level (Williams et al., 2009; Ozawa et al., 2010; Weber and Harrison, 2019), and modify HRV. Reducing light exposure through eye-mask and covering incubators/cribs can induce a QS with a stable respiratory rate (Shiroiwa et al., 1986; Venkataraman et al., 2018). For sound regulation, positive stimuli, such as human voice, rather than forcefully excluding stressful events, may represent a better option (Weber and Harrison, 2019). The use of tools, such as earmuffs, showed ambiguous results: some studies revealed higher oxygen saturation and quieter sleep, whereas other studies proved higher stress and lower HF (Zahr and de Traversay, 1995; Aita et al., 2013; Almadhoob and Ohlsson, 2020).

The incubator can influence newborns’ HRV also through the emission of electromagnetic fields, which indeed correlate with adverse health consequences in infancy (Li et al., 2012). When the incubator power is turned on, LF/HF increases, whereas HF decreases (Bellieni et al., 2008). Positioning the newborn as far away as possible from the incubator power can limit and reverse these effects (Bellieni et al., 2008; Passi et al., 2017).

From the HeRO experience, we also know that some drugs (i.e., dexamethasone, paralytics, anesthetics, and anticholinergics), surgery, and initiation of mechanical ventilation can significantly alter various HRV metrics (Fairchild and O’Shea, 2010; Fairchild, 2013).

If all these factors can impact HRV, thereby biasing its measurement, a real-time monitor could overcome these biases by recording how HRV changes before, during, and after the occurrence of these factors. Again, ML algorithms could help cope with these confounding factors: by weighting and integrating their effects, such technology could help reduce false alarms, exactly as those algorithms that can detect artifacts due to baby’s movements (Ostojic et al., 2020).

### The Clinical Usefulness of Real-Time HRV to Monitor Newborns

As with HR and SpO_2_, HRV could be monitored in real-time to provide information about the infant’s current conditions.

Real-time HRV helped identify stress-related behaviors, which are difficult to visually recognize: for example, LF increased during stress behaviors, whereas HF increased with self-consoling behaviors (Gardner et al., 2018). Moreover, real-time HRV monitoring—as revealed by changes in short- and long-term metrics, such as RMSSD, LF/HF, SampEn, and DFA α1 (but also SDNN, LF, HF, SD1, SD2, ApEn, and DFA α2) or in the newborn infant parasympathetic evaluation (NIPE)—correlated with nociceptive events, such as heel stick (Weissman et al., 2012; Butruille et al., 2015).

Derived from short-term HRV metrics related to PNS and reflecting HFn variations, the NIPE aims to assess in real-time the level of the newborn’s comfort and nociception (Butruille et al., 2015). Although it failed to correlate with pain scales measuring acute neonatal pain (Cremillieux et al., 2018) and with hemodynamic change following endotracheal intubation in children aged 1–24 months (Zhang et al., 2019), the NIPE succeeded in evaluating prolonged neonatal pain (Buyuktiryaki et al., 2018) and the balance between nociception and antinociception (Weber et al., 2019; Zhang et al., 2019).

Studies that continuously recorded HRV for more than 24 h, or that reviewed those recordings if available, led to discovering that HRV alteration can precede adverse outcomes even by 24 h (Griffin and Moorman, 2001; Stone et al., 2013; Kumar et al., 2020). Many of the studies about the predictive power of real-time HRV, in particular, in case of infections, relied on the HeRO monitor (Andersen et al., 2019; Kumar et al., 2020). The NIPE technology represents another attempt of introducing real-time HRV monitoring in NICUs, but it needs further evidence (Weber et al., 2019; Zhang et al., 2019).

It was revealed that HRV measured with the HRC index decreased 6 h before medical NEC and even 16 h before surgical NEC, thus also indicating the severity of the newborn’s condition (Stone et al., 2013). Abnormal heart rate characteristics (HRC) correlated with a several-fold increase in the risk of sepsis, urinary tract infections, and death in the day and week next to alteration (Griffin et al., 2005), and it could also precede the occurrence of SIDS (Zhao et al., 2016). In infants aged 28–35 weeks, low HF (less than 4.68 ms^2^) was found to correlate with the incidence of NEC stage 2+ (Doheny et al., 2014).

In a recent study, HRV combined with respiratory variability analysis changed significantly during the 24 h before the diagnosis of late-onset sepsis, especially in the last 6 h where real-time monitoring showed signals of cardiorespiratory instability. The reduction in RMSSD, accompanied by a decrease in average acceleration response (AAR, a robust index evaluating the heart capacity to increase its rhythm) and an increase in respiratory variability-related ApEn, predicted late-onset sepsis the most (Joshi et al., 2020).

The HeRO monitor, allowing NICU professionals to continuously observe the HR characteristics variation, reduced relative mortality due to sepsis even more than 20% (Fairchild, 2013; Kumar et al., 2020), especially when birth weight was lower than 1,000 g (Moorman et al., 2011).

A system that assesses real-time HRV can thus have significant clinical implications. Such a system could display both short- and long-term HRV metrics, thus augmenting HRV predictive usefulness (Voss et al., 2013) and overcoming the limits of short-time metrics, which are highly sensitive to artifacts (Stapelberg et al., 2017). Due to being non-invasive, real-time HRV would also be preferable to other assessment procedures that can cause discomfort to newborns and take time to be performed (De Jonckheere and Storme, 2019).

However, what could be the best way to make HRV an innovative tool that could augment the newborn’s management?

## The Future Perspectives for the Clinical Use of HRV in the NICU

### Technological Innovation to Monitor HRV in Real-Time

HRV monitoring is more accessible and less invasive than other diagnostic and predicting methods, such as blood sampling for measuring inflammatory cytokines and predicting sepsis (Fairchild, 2013). Real-time HRV monitoring could, thus, represent a significant step forward to improve baby care, especially when clinical decision-making is based on the sole experience of NICU professionals (Mowery et al., 2011; Oliveira et al., 2019a; Weber and Harrison, 2019).

Relying upon the instruments already present in NICUs is paramount (Rajalakshmi et al., 2019); in particular, due to the advantages over ECG, PPG could be the instrument to choose, although PPG-based computation can suffer from precision issues compared with ECG-based computation (Kevat et al., 2017; Pernice et al., 2019b; Henry et al., 2020).

For the sake of simplicity and for attracting clinicians to the use of HRV (King, 2020), the first step could be introducing in NICUs a PRV/HRV monitor that shows a single metric able to correlate with the infants’ clinical conditions or development. Although the literature shows that the combination of more metrics can perform better than the use of single metrics (as detailed in previous sections), the literature also shows that single metrics can display good performance (more details in the next sections).

In infants, RMSSD correlated with several outcomes in both the short and the long term: ANS stage of development in the case of prematurity (Hoyer et al., 2009; Lucchini et al., 2016; Aye et al., 2018; Schneider et al., 2018), late-onset sepsis (Joshi et al., 2020), extubation success (Latremouille et al., 2018), recovering after admission in intensive care units (Marsillio et al., 2019), pain behavior (Weissman et al., 2012), short-term neurological development when measured during low blood pressure episodes (Semenova et al., 2018), and later neurological development at 2 years (Dimitrijević et al., 2016). RMSSD showed also to change according to the incubator temperature, thus revealing how infant’s HRV changes due to environmental temperature (Stéphan-Blanchard et al., 2013).

RMSSD seems a reliable index of HRV modulation by vagal activity (Shaffer and Ginsberg, 2017) and has good statistical properties (Task Force of the European Society of Cardiology the North American Society of Pacing Electrophysiology, 1996), and in adult studies, it was associated with adult health more than immune, inflammatory, and metabolic blood markers (Jarczok et al., 2015).

The good relationship with vagal activity makes RMSSD particularly interesting for neonatologists, for three main reasons: (a) the PNS develops during the last weeks of gestation—when preterm birth can occur—and after birth (Schneider et al., 2018; Patural et al., 2019), (b) the PNS activity correlates with the infant’s conditions and development (Mulkey and du Plessis, 2019), and (c) since vagal activity correlates positively with inflammation regulation (Fairchild and O’Shea, 2010), RMSSD could help detect newborn’s inflammation (as shown in animal fetuses, Frasch et al., 2016). The relation of RMSSD with vagal-related HRV modulation could help clinician in easily interpreting RMSSD changes: the lower the RMSSD, the lower the modulation by the vagal activity, and thus, the worse the infant’s conditions.

As a time-domain metric, RMSSD has the advantage over frequency-domain metrics of being a more “direct” measure: RMSSD is obtained by merely calculating the root mean square value of successive time differences between heartbeats (Shaffer and Ginsberg, 2017). Hence, it does not suffer from the conceptual biases that could arise with frequency-domain metrics due to choosing a frequency threshold not validated in humans (Hayano and Yuda, 2019).

Therefore, the NICU real-time monitor could report RMSSD tracking in the previous 24 h, 4–5 min, and also 10–30 s—ultra-short RMSSD recordings are as reliable as short ones (Munoz et al., 2015). The monitor could be equipped with an alarm that activates when the actual RMSSD value changes over a threshold from the newborn’s past mean value. The alarm could activate when RMSSD decreases—lower modulation by vagal activity and, thus, risk of worse outcomes—but also when it rises significantly. For instance, an increase in RMSSD could represent pathological conditions, such as severe unconjugated hyperbilirubinemia (in association with low minimum HR) (Özdemir et al., 2018), or the occurrence of an antenatal/intrapartum hypoxia–ischemia that could lead to brain injury (Frasch, 2018; Yamaguchi et al., 2018).

To make HRV a gold standard, however, there is the need to deepen the knowledge about RMSSD or any other metric: more robust studies on larger samples are needed (Stéphan-Blanchard et al., 2013; De Jonckheere and Storme, 2019) as studies still show contradictory findings that merit careful analysis (Wang et al., 2016; Joshi et al., 2020).

A further crucial step is to integrate HRV signals with other vital signs, such as SpO_2_ and blood pressure, to improve its predictive power and reduce mortality (Sullivan et al., 2018; Kumar et al., 2020). Even further is the integration of information about the infant’s gestation, labor, and perinatal period is essential: since NICU professionals have to deal with complex pathologies, including SIDS (Siren, 2017), technological tools that can elaborate all these types of information could become paramount to improve clinical care (Hemphill et al., 2011).

Therefore, in parallel with introducing a real-time HRV monitoring system, there might be the possibility to start using the tools already present in NICUs to collect a large amount of data about all the babies’ vital signs (Kumar et al., 2020). This data could then be analyzed by ML algorithms, whose results the bedside monitors would display as one or more metrics, graphs, or models (Bravi et al., 2011; Hemphill et al., 2011; Seely et al., 2011).

Indeed, several fields in medicine share this need of collecting and using a large amount of data to improve patient care. An example is the neurocritical care where, as any other intensive care unit, “extreme complexity reigns”: continuous multimodal monitoring is vital to guide an efficient decision-making process with the purpose of preventing complications and warranting survival, especially in case of emergency (Hemphill et al., 2011; Lara and Püttgen, 2018).

However, this means revolutionizing the NICU itself. First, the data must be stored and automatically controlled for artifacts. Then, all the medical devices have to communicate and to be synchronized with each other to assure the collected data are coherent; they could also connect to a central computer/hub that autonomously synchronizes the data process. Synchronization is essential: otherwise, data interpretation would become flawed. Lastly, clinicians could add laboratory values, imaging results, and medical documentation to the patient’s record (Hemphill et al., 2011).

Advanced bioinformatics could then follow with the use of the many available techniques of data mining coming from statistics, ML, and AI. For example, ML or Deep Learning approaches can be used, including the ones based on decision trees and neural networks, especially dynamic Bayesian neural networks, which capture well non-linear phenomena with complex multimodal relationships. Based on the big-data of other patients, such approaches could elaborate the past and present information of the actual patient to predict the evolution of the clinical conditions. Moreover, they could visualize this prediction in more useful ways (e.g., from numerical probability to specific types of graphs) than raw data or simple statistical metrics (Hemphill et al., 2011).

Multimodal monitoring and the efficient application of data mining algorithms in medicine have yet to grow. In fact, many obstacles (e.g., the availability of resources) may hinder such development, but the complexity of intensive care units makes a strong call to action, and the stakeholders in the healthcare systems must be aware of the opportunities informatics could give to clinical care (Hemphill et al., 2011; Lara and Püttgen, 2018).

The stakeholders must also know that it is paramount to establish tight collaborations between the obstetric, neonatal, and pediatric departments: sharing data about patients’ health and medical devices among these departments would elicit high-quality research that would help clinicians to correlate neonatal outcomes to their fetal development and past history, thus truly improving neonatal care—otherwise, how would it be possible to treat neonatal pathologies ignoring their fetal origin?

In the next sections, it is argued as HRV analysis could indeed represent a useful tool to help both in decision-making and in monitoring the infant’s condition.

### HRV as an Innovative Tool in Neonatal Decision-Making Intervention

Because various metrics, such as RMSSD, relate to HRV modulation by vagal tone, which plays a significant role in infant’s condition, HRV monitoring could give reliable information about the infant’s level of stress (Mulkey and du Plessis, 2019).

Indeed, since LF and HF inversely correlated with salivary cortisol levels in both healthy and NICU admitted newborns (Hashiguchi et al., 2020), real-time HRV monitoring could assess the actual stress level of newborns. Moreover, since stress- and pain-related behaviors in newborns are difficult to discriminate, real-time HRV could represent a valuable tool to assess both neonatal stress and pain (Cremillieux et al., 2018; Zhang et al., 2019).

As neonatal stress and pain can adversely influence neurodevelopment and brain maturation, as well as future health and longevity (Holsti et al., 2006; Reynolds, 2013; Pillai Riddell et al., 2015; Simeoni et al., 2018), every procedure that can improve the newborn’s condition is paramount, especially in case of prematurity or pathological conditions. Knowing when and how to apply such procedures is even more essential: a real-time HRV monitor that gives information about the actual newborn’s level of stress could elicit NICU professionals to promptly adjust the NICU environment to improve the newborn’s wellbeing and comfort.

Real-time HRV would help nurses to adopt correct behaviors for reducing visual and auditory overstimulation: in a complex environment, such as the NICU, it is indeed difficult to know exactly when to perform these procedures (Aita and Goulet, 2003).

Based on the value of the index shown by the HRV monitor, NICU staff could increase or decrease the temperature of the incubator, change the illumination intensity or type, and introduce positive stimuli: for instance, human voice or songs, even recorded, cloths with parent-scent, eye contact, and gentle touch (Ozawa et al., 2010; Stéphan-Blanchard et al., 2013; Weber and Harrison, 2019).

Real-time HRV could also help choose between “positive” procedures, such as facilitated tucking—manually supporting the baby by holding the upper and lower extremities in flexion—plus human voice or skin-to-skin contact between the mother and the newborn: these procedures, if well performed, should increase the level of newborn’s comfort, and a HRV monitor could be able to show their actual benefit (Alexandre et al., 2013; Butruille et al., 2017; Marvin et al., 2019).

As shown in sections “The ANS Development and Its Relationship With HRV” and “Factors That Can Alter HRV Assessment”, HRV metrics could change according to environmental factors. However, this knowledge must be deepened, and the faster way to achieve this result could be introducing HRV monitoring in as many NICUs as possible and by collecting a large amount of data, from which ML applications could extrapolate models or metrics suitable and clinically-sound for the above-mentioned conditions.

A real-time HRV monitor could help understand when newborns need analgesic medications to withstand better invasive and noxious procedures (Weber et al., 2019) or adjuvant neuroprotective therapies in case of HIE—infants who subsequently died due to HIE showed lower LF and higher HF—(Massaro et al., 2014), and it could also help choose better how to administer surfactant in case of respiratory distress syndrome—the NIPE index showed different values according to the technique used (Okur et al., 2019).

In the same way, HRV could guide the choice among various respiratory supports post-extubation, besides predicting the outcome of extubation. Indeed, RMSSD, SDNN, pNN50, TP, and VLF were higher during non-synchronized non-invasive ventilation than during nasal continuous positive airway pressure (CPAP) in extreme preterms who were reintubated a few hours later (Latremouille et al., 2018). Such difference was not found in infants who showed successful extubation, even though every HRV metric analyzed was higher (but not significantly) during ventilation than nasal CPAP. Therefore, the authors speculated that those variations could correlate with the failure in extubation, and that non-invasive ventilation could better modulate the ANS of less stable infants than nasal CPAP (Latremouille et al., 2018). HRV measured as SDRR was also found to increase during high flow nasal cannula, but not during nasal CPAP, in extremely preterm infants who succeeded in being extubated (Latremouille et al., 2019).

### HRV as an Index to Monitor Clinical Improvement

To be an all-round innovative tool, real-time HRV should not only predict future outcomes with good accuracy but should also provide useful information to monitor the current condition of the newborn.

The question is: could HRV help understand whether the baby’s condition is deteriorating or improving?

A real-time HRV monitoring system could give a clear answer. Besides, this is another field in which more data and the use of ML software could dramatically increase the clinical usefulness of HRV, and thus another reason to introduce HRV monitors in NICU and to integrate their recordings with the infant’s development over time.

HRV—measured through several metrics including HRC index, DFA α_S_, RMS_S_, RMS_L_, LF, and HF—could change greatly in case of pathological conditions, such as congenital heart disease, cardiac failure in persistent ductus arteriosus, respiratory deterioration with apnea, respiratory distress syndrome, inflammation, hyperbilirubinemia, respiratory syncytial virus, and sepsis. Indeed, inflammatory cytokines (e.g., IL-6, IL-8, and IL-13), bacterial toxins, and free bilirubin could pass through the immature brain–blood barrier and exert neurotoxic activity, thus negatively influencing the nervous system. The same negative effect can be induced by the cardiorespiratory pathologies (van Ravenswaaij-Arts et al., 1991; Prietsch et al., 1992; Stock et al., 2010; Raynor et al., 2012; Sullivan et al., 2014; Uhrikova et al., 2015; Al-Shargabi et al., 2017; Mulkey et al., 2020).

Since HRV modifications correlated with inflammatory cytokine levels (Al-Shargabi et al., 2017), real-time HRV could be a useful marker of clinical acuity: indeed, HRV alterations—a decrease in RMS_S_, RMS_L_, DFA α_S_, LF, HF, and TP or an increase in HRC index—correlated with the pathology severity (Sullivan et al., 2014; Al-Shargabi et al., 2018). Variations in LF, HF, and LF/HF correlated with HIE severity, although more studies are needed to resolve conflicting results between studies (Andersen et al., 2019). In a recent paper on a pediatric population, compared with the time of admission in the intensive care units, RMSSD, SDNN, and pNN50 continuously rose as the time of discharge approached, that is, as the infants recovered (Marsillio et al., 2019).

The correlation between HRV alterations, inflammation, and inflammation-related pathologies can be better understood through the cholinergic anti-inflammatory pathway (CAP). Briefly, the CAP begins with the afferent vagus nerve that detects inflammatory cytokines or bacterial toxins and transmits these signals to several brain nuclei, which then induce an anti-inflammatory response through the release of acetylcholine. This response involves complex interactions between both ANS branches and plays a paramount role in finely tuning the immune response and avoiding tissue damages in case of inflammation (Garzoni et al., 2013; Bonaz et al., 2017).

The CAP can fail to properly function in case of ANS dysfunction, but also due to pathogens and inflammation: pathogens can rapidly activate but desensitize the CAP (i.e., alter cholinergic receptors) (Fairchild et al., 2011), whereas prolonged inflammation can induce apoptosis in the brainstem vagal nuclei (Fritze et al., 2014). As a result, HRV decreases, and adverse outcomes can occur: indeed, low vagal activity and reduced CAP efficiency correlate with increased inflammation, morbidity, and mortality, in both infants and adults (Garzoni et al., 2013; Bonaz et al., 2017).

In fetuses, the CAP, although at the early stage of maturation, might also modulate local and systemic inflammation (Garzoni et al., 2013). Indeed, animal studies suggested that in ovine fetuses, CIMVA applied to fHRV found several metrics that uniquely predicted, 1.5 days in advance, M1 and M2 macrophages activation and occludin expression (i.e., increased leakiness) in the terminal ileum (Liu et al., 2016). Therefore, if cytokines and toxins can alter HRV through the CAP, it might be intuitive why HRV analysis can inform about the global health of the infant and predict future inflammation-related development.

HRV modifications could also reflect the effect of treatments, such as ventilation, antibiotics, and phototherapy. To date, a clear correlation is still lacking, and better research is needed, although initial evidence is available (Sullivan et al., 2014; Uhrikova et al., 2015; Al-Shargabi et al., 2017; Latremouille et al., 2018). For instance, as RMS_S_, RMS_L_, DFA α_S_, LF, HF, and TP decreased around 48 h before NEC occurred, the same metrics recovered to their baseline values during the first 60 h after NEC diagnosis (Al-Shargabi et al., 2018). Monitoring HRV in the 60 min prior to extubation could help predict which babies will have an adverse outcome, since they could show much lower TP, LF, HF, LF/HF, and VLF than babies who will succeed in being extubated (Kaczmarek et al., 2013).

Other indications about the clinical usefulness of HRV, and real-time monitoring, come from the studies on HIE severity and ANS in infants. During therapeutic hypothermia for HIE, lower RMS_S_, RMS_L_, DFA α_S_, and LF and higher HF indicated a higher risk of secondary energy failure, brain injury, and death, thus helping in identifying the infants who failed to respond to hypothermia and needed adjuvant neuroprotective therapies (Govindan et al., 2014; Massaro et al., 2014; Metzler et al., 2017). The analysis of DFA α_S_, RMS_S_, and RMS_L_ could also prove useful to discriminate among different types of brain injury, such as white matter injury, watershed stroke, basal ganglia injury, or global injury (Metzler et al., 2017). As shown with fHRV analysis, HRV monitoring, especially during labor, could also predict which newborns are at risk of developing neuroinflammation and, thus, brain injury—neuroinflammation is correlated to severe acidemia, and fHRV monitoring could detect it with 1 h of advance. Since therapeutic hypothermia is poorly applied, either because often the brain injury has already developed or because neonatologists lack tools to properly recognize which newborns require hypothermia, HRV monitoring could overcome this predicament (Frasch et al., 2014; Xu et al., 2014; Gold et al., 2019).

Real-time HRV is shown to be useful for monitoring how infants react to routine-care procedures, such as diaper changes or pupil examination, and for revealing neurological abnormalities. Indeed, infants with HIE and impaired ANS—defined as at least one alteration in LF, HF, DFA α_S_, RMS_S_, or RMS_L_—showed HR, blood pressure, and cerebral blood flow alteration during those procedures. Moreover, the more the number of altered HRV metrics, the worse the outcome, i.e., moderate severe brain injury or death (Campbell et al., 2018).

Real-time HRV could also help mothers and fathers to monitor the interaction with the baby: using a feedback and feedforward process, parents could see how their actions affect their baby (Van Puyvelde et al., 2019a, b; these studies, however, examined how touch influenced RSA and RR intervals, not specific HRV metrics).

Furthermore, real-time HRV could help control how the infant’s condition progresses in different positions (supine, prone, or side-lying). This monitoring would be useful, for example, to balance the need for proper oxygenation and avoiding adverse outcomes, such as SIDS. Infants who succumbed to SIDS showed lower RSA during all sleep stages (Kluge et al., 1988) and altered Poincaré plots compared with controls (Schechtman et al., 1992). Those infants showed also higher basal HR during QS (Kelly et al., 1986), lower HF, and higher LF before pre-apneic sighs than those infants who did not die from SIDS (Franco et al., 2003).

Newborns who develop SIDS seem to show an autonomic instability that impedes them from facing a life-threatening event (Elhaik, 2016; Zhao et al., 2016). Real-time HRV could help prevent this adverse scenario. Besides, due to the uncertainty still surrounding SIDS—for instance, infants who succumb to SIDS could even show the same RSA and HR, but higher LF than controls (Gordon et al., 1984)—, continuous monitoring data analyzed by AI algorithms could help shed more light on SIDS occurrence by giving useful information about the newborn’s ANS condition.

Speaking of which, an ML algorithm based on boosted decision trees increased the predictive power of HRV during episodes of low mean blood pressure in preterm infants with a GA between 23 and 31 weeks. Although good results were obtained even with single metrics—among time, frequency, and non-linear ones, RMSSD gave the best area under the curve (0.87)—combining all the metrics through the mentioned algorithm gave an area under the curve of 0.97 for predicting short-term neurological outcomes (i.e., grade III/IV intraventricular hemorrhage or cystic periventricular leukomalacia, NEC, bronchopulmonary dysplasia, infection, and retinopathy) (Semenova et al., 2018).

Lastly, real-time HRV monitoring could give information about the neurological development. Indeed, newborn’s HRV predicted the occurrence of neurological abnormalities—cerebral palsy, language or mental retardation, vision or hearing disability, or attention disorders—at 2 years (Thiriez et al., 2015; Dimitrijević et al., 2016). Twenty-four hour RMSSD, SDANN, and SDNN were particularly good predictors, respectively, 88.9, 83.3, and 83.3% specificity and 100% sensibility using the threshold values of 17, 38, and 47 ms—and also improved the prognostic value of general movements assessment: among newborns with poor repertoire general movements, those with higher RMSSD, SDANN, and SDNN showed better neurological development (Dimitrijević et al., 2016).

### HRV Potential Usefulness in Middle- and Low-Income Realities

HRV could be a useful non-invasive tool to assess infant’s conditions and to reduce the risk of adverse outcomes and, as the HeRO monitor showed, mortality from sepsis. This last result is worth noting since neonatal sepsis remains one of the major causes of neonatal mortality, together with prematurity and birth-related complications, and this holds especially in middle- and low-income countries (WHO, 2016).

Indeed, only 15 in 48 Asian and African countries reported having an indicator about sepsis management in their Health Management Information Systems (WHO, 2017). In countries, such as South Africa and India, about 1 infant in 35 dies before the first birthday, even more than ten times compared with developed countries, such as Japan, Sweden, or Italy (about 1 in 350–500) (OECD, 2020). Closing this large gap by reducing worldwide neonatal mortality to less than 1 infant in 100 was deemed by the WHO as one of the top priorities in 2015 (WHO, 2016). Of notice, the first 28 days after birth shows the highest mortality risk (United Nations Inter-agency Group for Child Mortality Estimation (UN IGME), 2019).

The studies that investigated the reasons behind the difference in mortality highlighted that the barriers to efficient care were twofold. On the one hand, people tend to delay seeking care due to lack of knowledge about neonatal health, sociocultural behaviors (e.g., relying on traditional practices), and several concerns about the cost of healthcare, the attitude of NICU staff, and the lack of appropriate medical equipment (Watson et al., 2020). On the other hand, obstetrics and neonatal evidence-based practices fail to be introduced due to lack of financial resources, workforce capacity, and specific training in case of emergency. The effect is a low-quality screening for pathological conditions, such as IUGR or sepsis, which can lead to severe neonatal health problems and even death (Hoyer et al., 2017; Otieno et al., 2018).

To date, real-time HRV monitoring cannot solve all these complex problems, but it could surely address financial- and efficiency-related obstacles. Indeed, HRV monitors based on PPG, contactless vPPG, or even BSG could be low-cost and easy-to-use devices (Sun et al., 2012; Sun and Thakor, 2016; Joshi et al., 2018) that allow midwifery and NICU staff to efficiently monitor the newborn’s development, screen for pathological conditions, and choose the best treatment (Hoyer et al., 2017). A recent paper showed that the HeRO monitor could also help reduce, even if a modest effect was shown, the use of antibiotics and blood culture tests for suspicion of sepsis in the first 120 days of life (King, 2020).

Moreover, if relying on big-data and the Internet, the HRV monitors could even be simple systems that monitor the babies, send information to a central hub (e.g., through the Cloud), and receive the corresponding elaboration, thus having the same efficiency of HRV monitors in high-income countries (Werth, 2019).

## Conclusion

The present paper reviewed the use of HRV in the neonatal field and the utility of real-time HRV monitoring to assess the newborn’s clinical conditions, showing that several metrics and computed metrics change in conjunction with stress-/pain-related behaviors, inflammation, pathological conditions, such as cardiac failure, respiratory distress syndrome, hyperbilirubinemia, NEC, and sepsis, and neurological development.

The paper also reviewed the NICU technology to evaluate how to measure real-time HRV efficiently. Indeed, a system based on PPG could be the optimal solution due to being low-cost, easy-to-use, and non-invasive, although PPG-based computation seems less precise than ECG-based computation. Therefore, future studies will have to carefully assess if the outcomes reviewed in this paper might be influenced by this difference in precision between PPG and ECG.

In the next decade, introducing real-time HRV in NICUs would be a great step forward in the improvement of neonatal care, especially if supported by the advancements in bioinformatics, which could easily extrapolate accurate predicting models from all the data collected in the NICUs, although several concerns and limitations have to be overcome before fully implementing the system into a daily NICU routine care.

## Author Contributions

MC, FCe, AC, NB, and AM contributed equally to the conceptualization and to the writing of the manuscript. DB, FCa, and CC revised and edited the final draft of the manuscript. All authors approved the final manuscript as submitted and agreed to be accountable for all aspects of the work.

## Conflict of Interest

DB was employed by the company BioTekna. The remaining authors declare that the research was conducted in the absence of any commercial or financial relationships that could be construed as a potential conflict of interest.

## Abbreviations

AI: artificial intelligence
AMP: amplitude fluctuations
ANS: autonomic nervous system
AS: active sleep
BSG: ballistography
CAN: central autonomic network
CAP: cholinergic anti-inflammatory pathway
CIMVA: continuous individual multiorgan variability analysis
CNS: central nervous system
COMP: complexity
CPAP: continuous positive airway pressure
CTG: cardiotocography
ECG: electrocardiogram
fABAS: fetal autonomic brain age scale
fECG: fetal electrocardiogram
fHRV: fetal heart rate variability
fMCG: fetal magnetocardiography
FSE: fetal scalp electrode
GA: gestational age
HeRO: Heart Rate Observation system
HIE: hypoxic–ischemic encephalopathy
HR: heart rate
HRC: heart rate characteristics
HRV: heart rate variability
IUGR: intrauterine growth restriction
ML: machine learning
NEC: necrotizing enterocolitis
NIPE: newborn infant parasympathetic evaluation
PNS: parasympathetic nervous system
PPG: photoplethysmography
QS: quiet sleep
PNS: parasympathetic nervous system
PRSA: phase rectified signal averaging
PRV: pulse rate variability
RSA: respiratory sinus arrhythmia
SIDS: sudden infant death syndrome
SNS: sympathetic nervous system
SpO_2_: partial oxygen saturation
vPPG: non-contact video-photoplethysmography.
